# Reducing ligation bias of small RNAs in libraries for next generation sequencing

**DOI:** 10.1186/1758-907X-3-4

**Published:** 2012-05-30

**Authors:** Karim Sorefan, Helio Pais, Adam E Hall, Ana Kozomara, Sam Griffiths-Jones, Vincent Moulton, Tamas Dalmay

**Affiliations:** 1 School of Biological Sciences, University of East Anglia, Norwich, NR4 7TJ, UK; 2 School of Computing Sciences, University of East Anglia, Norwich, NR4 7TJ, UK; 3 Faculty of Life Sciences, University of Manchester, Manchester, M13 9PT, UK

**Keywords:** Next generation sequencing, MicroRNA, Small RNA, MiRBase, Expression profile, Deep sequencing, T4 RNA ligase

## Abstract

**Background:**

The use of nucleic acid-modifying enzymes has driven the rapid advancement in molecular biology. Understanding their function is important for modifying or improving their activity. However, functional analysis usually relies upon low-throughput experiments. Here we present a method for functional analysis of nucleic acid-modifying enzymes using next generation sequencing.

**Findings:**

We demonstrate that sequencing data of libraries generated by RNA ligases can reveal novel secondary structure preferences of these enzymes, which are used in small RNA cloning and library preparation for NGS. Using this knowledge we demonstrate that the cloning bias in small RNA libraries is RNA ligase-dependent. We developed a high definition (HD) protocol that reduces the RNA ligase-dependent cloning bias. The HD protocol doubled read coverage, is quantitative and found previously unidentified microRNAs. In addition, we show that microRNAs in miRBase are those preferred by the adapters of the main sequencing platform.

**Conclusions:**

Sequencing bias of small RNAs partially influenced which microRNAs have been studied in depth; therefore most previous small RNA profiling experiments should be re-evaluated. New microRNAs are likely to be found, which were selected against by existing adapters. Preference of currently used adapters towards known microRNAs suggests that the annotation of all existing small RNAs, including miRNAs, siRNAs and piRNAs, has been biased.

## Introduction

Improving the in vitro activity of nucleic acid-modifying enzymes has been a vital driver for molecular biology research, enabling technological advances in cloning, sequencing, forensic science, diagnostics and drug development. Much effort has therefore gone into understanding their function. In many cases these enzymes have evolved to recognise specific features to attain specificity, but a method to comprehensively describe these specificity determinants is lacking.

The characterisation of these determinants is important both to understand biological processes and to modify features for purposes of molecular manipulation. For example, DNA polymerases have been modified to improve fidelity and inhibitor resistance [1,2]. RNA ligases have also been studied in detail: thermophylic forms have been identified [3], and modifications to accept only adenylated RNAs have been made [4-6]. These new forms of RNA ligase were instrumental in the development of new protocols for the small RNA cloning required for next generation sequencing (NGS). Currently, identifying the functional determinants of their substrates has been based on low-throughput experiments.

Several innovative approaches using NGS to test millions of molecules in parallel have been developed to study protein function [7,8]. Most notably high-throughput sequencing-fluorescent ligand interaction profiling (HiTS-FLIP) is a technique for measuring quantitative protein DNA binding [8]. NGS has also been combined with SELEX, which uses randomised oligonucleotides to identify ligands for proteins [9] or transcription factor binding sites [10]. It was also used to establish the fitness landscape of a catalytic RNA [11] and to compare the bias of different approaches to sequence mRNA fragments [12].

**Figure 1:**
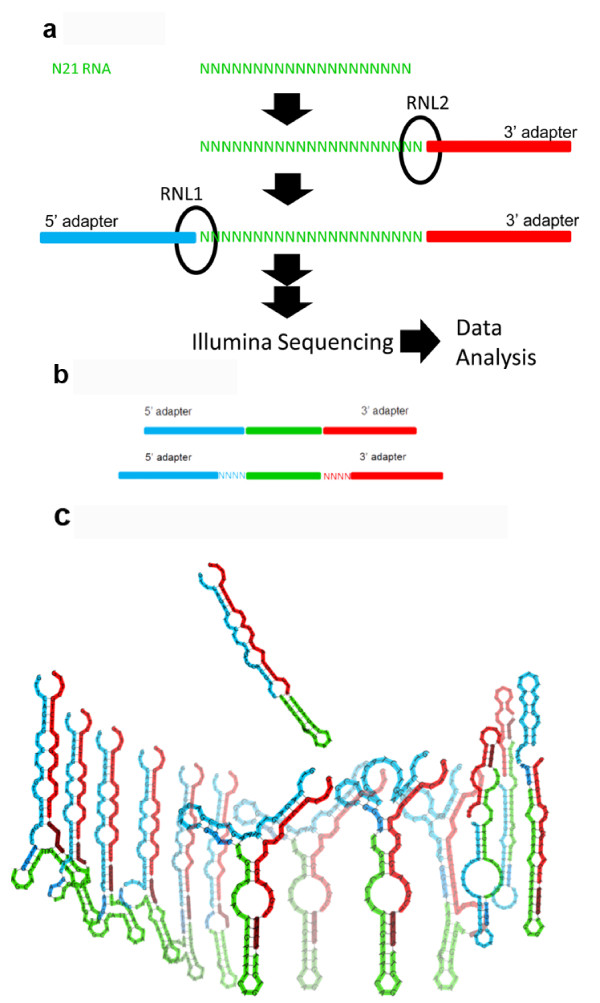
**Scheme depicting the experimental approach and HD adapters**. **a** Data were generated to analyse the sequence preferences of T4 Rnl1 and T4 Rnl2 using a degenerate RNA library (N21 RNA). **b** HD adapters include degenerate tags at the end of the adapters that allow the formation of stable secondary structures for more sequences and reduce RNA ligase-dependent sequence bias. Panel (**c**) shows the structure of miR-29b with the Illumina adapters (*top*) and some of the structures formed by HD adapters (*bottom*). We found 1,031 distinct structures originating from 12,479 tag combinations.

We have developed a method to carry out functional analysis of nucleic acid-modifying enzymes using NGS. This method employs completely randomised oligonucleotide substrates such that all possible sequences are presumed to have similar concentrations, which we call degenerate libraries. We add the enzyme of interest to the degenerate libraries containing millions of different sequences and subject the resulting sample to NGS (Figure 1a). The enzyme preferences are revealed by the NGS results. We used this approach to characterise RNA ligase sequence preferences in order to investigate the potential for biases in small RNA (sRNA) NGS data sets.

sRNAs are a major group of gene regulators between 20 and 32 nucleotides in length (reviewed in [13]) There are several classes of sRNA that play important roles in gene regulation, with the Dicer generated microRNAs (miRNAs) being the most extensively studied [14]. Their expression levels can be measured by array hybridisation, quantitative PCR (qPCR) or NGS of cDNA libraries (reviewed in [15,16]). Arrays and qPCR methods are limited to characterising known miRNAs, and recent reports have suggested significant differences between technologies for quantifying miRNAs [17,18]. Indeed, significant sequencing biases for NGS of miRNAs have been reported [19-21]. The latest protocol for small RNA library generation requires ligation of an adenylated 3' adapter using a truncated form of T4 RNA ligase2 (Rnl2), followed by ligation of a 5' adapter using T4 Rnl1, although other protocols that use T4 Rnl1 for both ligations are also commonly used. The ligated product is reverse transcribed and then amplified by PCR [22].

Rnl1 and Rnl2 are two different families of RNA end-joining enzymes and have two distinct in vivo functions. Rnl1 repairs the virus-induced cleavage of the single-stranded (ss) anticodon loop in tRNA-Lys in *Escherichia coli*[23-26]. A SELEX type approach was used to show that Rnl1 prefers ss substrates [27]. Rnl2 is involved in RNA editing in eukaryotic trypanosomes and Leishmania [28,29]. The current thinking is that Rnl2 seals nicks in double-stranded (ds) RNA in keeping with its function in RNA editing of mRNA [30-32]. The phage T4 Rnl2 is commonly used in molecular biology. Although it can ligate both ds and ss RNA [32], it is not clear which structure is preferred, and its in vivo function is not currently known. A comprehensive understanding of RNA ligase substrate preferences would help in developing a method to reduce sequencing bias.

We used cDNA libraries generated through ligation of RNA molecules to survey the sequence preference landscape of Rnl1 and Rnl2 using degenerate libraries. This revealed important sequence preferences of these enzymes. This comprehensive analysis allowed us to develop a novel type of high definition adapter (HD adapter) (Figure 1b) that significantly reduces sequencing bias in biological samples. We demonstrate that the use of HD adapters increased the representation of low-abundance small RNAs and allowed new miRNAs to be identified. In addition, we use available data in miRBase [33], the global repository for miRNA sequences, to demonstrate that the dominant use of one NGS platform has biased miRNA research.

## Results

### RNA Ligase Characterisation Using NGS

**Figure 2:**
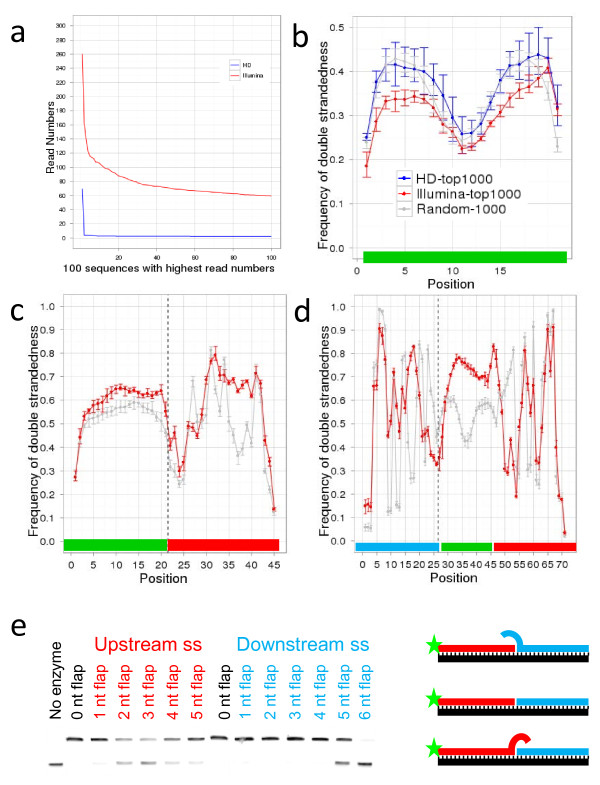
**Sequencing cDNA generated from N21 RNA libraries**. **a** Number of reads for the 100 most abundant sequences in the N21 libraries, prepared with Illumina (*red*) or HD adapters (*blue*). **b-d** Frequencies of predicted nucleotide base-pairing per position for N21 insert (**b**), N21 insert and 3’ adapter (**c**) and 5’ adapter, insert and 3’ adapter (**d**). In (**c**) and (**d**) *vertical dotted line* indicates ligation point. *Red line* denotes data obtained with Illumina protocol, *blue line* with HD protocol and *grey line* randomly generated sets of 21nt sequences. *Bars* indicate minimum and maximum values in all replicates. *Horizontal bars* at *bottom* indicate sequence region: *green*, insert; *red*, 3’ adapter; *blue*, 5’ adapter. For insert folding frequencies obtained with random sequences are more closely matched by HD data (R^2^ = 0.83) than by Illumina data (R^2^ = 0.60). **e** Comparison of T4 Rnl2 ligase activity on substrates with ss flaps of differing nucleotide lengths upstream or downstream of ligation site. In vitro ligation assay of RNA-DNA duplexes with either a nick (0NT) or ss flaps up- or downstream from the ligation site was carried out at 25°C for 30 min. Substrates with ss flaps >2nt in length upstream from the ligation site are inefficiently ligated. The diagram illustrates the position of the flaps, the fluorescein reporter group (*star*) and the backbone oligonucleotide (*black*). If ligation occurs the size of the nucleic acid attached to the fluorescein increases as visualised by 15% PAGE.

We subjected degenerate RNA libraries (100 pmoles N21 RNA and 3.4 pmoles N9 RNA) to the standard sRNA library preparation protocol, which uses 10 pmoles of the 3' adapter and 5 pmoles of the 5' adapter. The libraries were then sequenced on an Illumina GAII sequencer (Figure 1a). The count distribution obtained for the N21 RNA library was significantly different from the expected Poisson distribution (*χ*^2^-test, *p* < 10^-15^): for example, 58,956 sequences were found more than 10 times, instead of the expected one time (Figure 2a, Additional file 1: Table S1). The N9 RNA libraries also showed very strong bias that was significantly different from Poisson distribution (*χ*^2^-test, *p* < 10^-15^) (Additional file 2: Figure S1). Strikingly, despite obtaining ~18.5 million sequencing reads, only 42% of the 262,144 possible sequences were captured (109,998 distinct sequences). These data suggest that either the N21 and N9 RNA libraries were not equimolar for all possible sequences or that the ligases have preferences for particular sequences.

RNA ligase efficiency is dependent on the secondary structure context at the ligation site [5,34]. Therefore we investigated if the degree of secondary structure is correlated to the read number. We computationally folded all sequenced reads with the 3′ adapter sequence, and the minimum free energy (MFE) of the resulting RNA structures was computed for each molecule. The abundance of a sequence in the library showed a strong correlation to the value of MFE: sequences with more stable predicted structures are more abundant in the sequenced data (R^2^ = 0.48). This observation suggested that at least some of the bias was due to the ligation step and not because of the quality of the N21 and N9 RNA libraries.

To analyse the precise secondary structure preference of T4 Rnl1 used in the second ligation we generated a control data set by computationally folding 1,000 randomised 21mer oligonucleotides together with the 5′ and 3′ adapters. We then generated the secondary structure from the 1,000 most abundant sequences with the 5′ and 3′ adapter sequences. At the ligation site between the 5' adapter and N21 RNA only 25% of ligated RNA molecules were predicted to base pair compared to 49% in the control data set (Figure 2d). This very strong preference for ss ligation sites is consistent with the reported in vivo role of T4 Rnl1 and therefore supports the validity of this approach [26].

We repeated the analysis to investigate the secondary structure preferences of T4 Rnl2 used in the first ligation (Figure 2c). The results suggested that Rnl2 prefers to bind base paired nucleotides compared with the randomised data set. At the ligation site, 53% of the insert was base paired compared to 44% in the control data set. The data show a strong preference for ds nucleotides upstream of the ligation site but not downstream (Figure 2c).

We confirmed the high-throughput data using annealed oligonucleotides with either 3' ss flaps or 5' ss flaps (Figure 2e). A 1nt flap is tolerated either upstream or downstream of the ligation site (Figure 2e) although the ligation efficiency is reduced in time-course experiments (data not shown). However, longer ss flaps of 2-5nts upstream of the ligation site reduced ligation efficiency considerably. However, protruding ends of 1-4nts downstream of the ligation site are well tolerated, but a >4nt protruding end inhibits ligation (Figure 2e). In summary, these experiments validated the preference of T4 Rnl2 for ss nucleotides downstream of the ligation site but ds nucleotides upstream of the ligation site, as predicted by the sequencing data and in agreement with Hafner et al. [21].

### HD Adapters Reduce Ligation Bias

Based on these observations, we hypothesised that a population of degenerate adapters would average out the observed sequencing bias because the slightly different adapter molecules would form stable secondary structures with a more diverse population of sRNA sequences. This could allow: (1) the cloning of sRNAs that are normally not present in libraries generated by the traditional adapters and (2) the abundance of sequences to better the concentration of the sRNA in the sample. To test this hypothesis four random N nucleotides (A, C, G or U) were added to the 5′ end of the 3′ adapter and also to the 3′ end of the 5′ adapter. We named the resultant sequences high definition adapters (HD) (Figure 1b).

Using the 9 N RNA and 21 N RNA libraries we found that use of the HD adapters resulted in twice the sensitivity of the standard Illumina adapters. HD adapters captured 81% of possible sequences (213,188 distinct sequences) vs. 42% for Illumina adapters (109,998 distinct sequences) for the N9 libraries and read numbers were closer to the expected distribution for both N21 and N9 libraries (Figure 2a, Additional file 2: Figure S1). This indicates that although some of the bias described in the previous section may be due to un-equal representation of sequences in the N9 and N21 RNA libraries, most of the bias is the consequence of the ligation of specific adapters. We also show that the Illumina approach does not capture sequences that are predicted to fold back on themselves and that the HD approach is not biased in this way (Figure 2b). However the preference for sequences with secondary structures at the 3’ end was observed for both Illumina and HD adapters [35].

To analyse the effect of HD adapters on secondary structure preference of T4 Rnl1 we generated a control data set as described before (Figure 2c and d) and compared it to secondary structures of the 1,000 most abundant sequences with the 5′ and 3′ HD adapter sequences (Additional file 3: Figure 2b). We also repeated the analysis to investigate the secondary structure preferences of T4 Rnl2 used in the first ligation (Additional file 3: Figure S2a). These analyses showed that the secondary structures of the most abundant sequences obtained with HD adapters were more similar to the random set than the secondary structures of the most abundant sequences obtained with the Illumina adapters (Figure 2c and d).

The HD adapters represent a complex set of 256 adapters with 65,536 possible pairs. For the N9 RNA data, more than 60% of the sequences were captured with fewer than ten barcode pairs. This implies that individual adapter pairs had particular preferences for cloning a set of sequences (Additional file 4: Figure S3). This finding allows the design of unbiased adapter sets for multiplexing. An alternative application could be the manipulation of bias using adapters with specific tags, for example to preferentially sequence low abundance miRNAs associated with disease or to exclude highly abundant sequences that dominate the data.

Whilst this manuscript was in preparation three papers investigating the cause of bias in small RNA libraries were published. Two papers proposed a similar approach as HD adapters for reducing bias [19,20] and Hafner et al. [21] showed that secondary structures affect RNA ligase efficiency. Our more comprehensive data unify these works by demonstrating that HD adapters reduce bias through RNA ligase-dependent secondary structure dynamics and revealing the extent of bias using degenerate libraries and biological data sets.

### HD Adapters Reduce Sequence Bias in Libraries from Biological Samples

**Figure 3:**
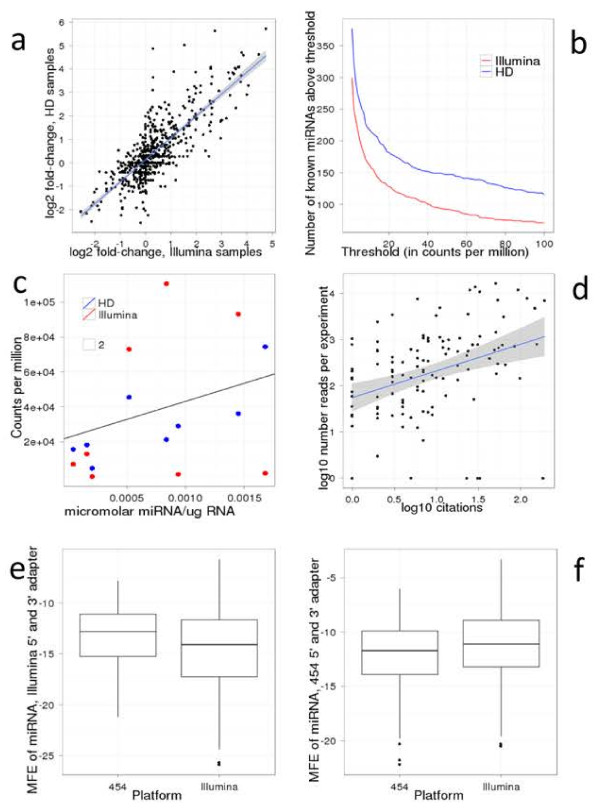
**cDNA library preparation protocols distort miRNA research**. **a** Comparison of change in miRNA level between wild-type and Dicer KO DLD cells obtained in Illumina (x axis) and HD samples (y axis). R^2^ = 0.62. **b** Number of known miRNAs found in DLD cells at different thresholds using Illumina or HD adapters. Regardless of chosen threshold, HD adapters identify more miRNAs. **c** Absolute quantification of eight known miRNAs (let-7i, miR-10a, miR-19b, miR-21, miR-25, miR-29b, miR-93, miR-375) obtained by Northern blot compared with number of times these miRNAs were sequenced using Illumina or HD adapters in DLD cell line. Data obtained with HD adapters correlates better with absolute quantifications (R^2^ = 0.70) than Illumina data (R^2^ = 0.12). **d** Number of PubMed citations and number of reads per experiment (data obtained from miRbase v17) of miRNAs conserved between mouse and human. MiRNAs with higher number of reads tend to be more extensively studied [R^2^ = 0.58, p-value < 10^(−15)^]. **e-f** Distributions of minimum free energy (MFE) of known human miRNAs concatenated with 5’ and 3’ adapter sequences. Using Illumina adapter sequences sRNA cloning kit V1.5 the set of miRNAs found by Illumina has lower average MFE than the set of miRNAs found by 454 (Wilcoxon test *p* = 0.01). We found the same result using the 3' adapter from sRNA cloning kit V1.0 (data not shown). **e** Conversely, using 454 adapter sequences average MFE is lower for set of miRNAs found by 454 (*p* = 0.07). **f** Analogous results for concatenation of miRNA only with 3’ adapter display a similar trend (see Additional file 5: Figure S7).

We next tested the HD adapters on biological samples to investigate their accuracy and sequence coverage. Libraries were generated using either Illumina or HD adapters from RNA of the DLD-1 colon cancer cell line and DLD-1 Dicer exon5 partial KO mutant cell line. Given that the biases are expected to be sequence specific, the same sequences in different samples will be subject to similar biases. Fold change expression analyses are therefore largely unaffected by these biases. We confirm that the fold change of miRNA expression between DLD-1 WT and DLD-1 Dicer KO were similar in libraries using HD and Illumina adapters (Figure 3a). Therefore both HD and Illumina adapters are valuable for identifying differentially expressed sRNAs.

The accurate quantification of sRNAs is crucial because researchers focus on miRNAs with high read numbers. We found that miRNAs with high read counts in miRBase were significantly more likely to be cited by the research community. (R^2^ = 0.25, p = 10^-15^, Figure 3d). This is not surprising because usually miRNAs that are highly expressed (i.e. have high read numbers) and show the strongest differential expression compared to another sample (control or another treatment or another tissue, etc.) are selected for in-depth, functional analysis. We ranked the miRNAs based on their normalised read number in DLD-1 cells using either HD or Illumina adapters (Additional file 6: Table S2). The most abundant miRNA in the HD adapter-generated libraries was miR-29b with more than 150,000 reads per million reads, which is almost twice as high as the next miRNA. Therefore it would be reasonable to choose miR-29b for further analysis if one was interested in the role of miRNAs in colon cancer biology. However, using the Illumina adapters, miR-29b was only the 29^th^ on the ranked list with 3,336 normalised reads, while the top four miRNAs had more than 100,000 normalised reads in that library (Additional file 6: Table S2). It is clear that miR-29b would not be chosen for further analysis based on the Illumina sequencing result. Furthermore, only five of the top ten most sequenced miRNAs using the HD adapters were also in the top ten most sequenced miRNAs using Illumina adapters. Therefore, the prioritisation of miRNAs for in-depth analysis could be highly dependent on the adapters used, at least for some samples. We used quantitative Northern blot analysis to demonstrate that libraries made with HD adapters accurately reflected cellular abundance of the sRNAs but libraries made with Illumina adapters did not (Figure 3c, Additional file 7: Figure S4). Not all miRNAs show such a dramatic difference in the two ranked lists (e.g. miR-93 and miR-10a were ranked second and third on the HD adapter list, and fourth and second on the Illumina adapter list, respectively) but the example of miR-29b illustrates that potentially many miRNAs were not chosen for functional analysis in previous studies.

Next we investigated the sequence coverage of HD adapters. We found that the HD protocol identified more than double the distinct sequences that mapped to the genome compared to a library prepared with Illumina adapters. HD adapters also captured approximately 25% more known miRNAs at any particular count threshold compared to Illumina adapters (Figure 3b).

HD adapters were also able to capture previously unidentified miRNAs. The miRCat algorithm [36] was used to identify 32 candidate miRNAs using HD or Illumina data (Additional file 8: Table S3) (predicted secondary structures and read numbers to pre-miRNAs are shown in Additional file 9: Figure S5). In addition to identifying 309 known miRNAs in this cell line the HD adapters were able to capture 26 new miRNAs. Five of these were also sequenced by the Illumina adapters, but there were only three new miRNAs, which were only captured by the Illumina adapters. The normalised read number of these 29 new miRNAs was at least 1.4 fold lower in the Dicer KO DLD-1 cell line, supporting that they are generated by Dicer. In addition, we searched deep sequencing data in miRBase and found reads matching the putative miRNA* sequences for all new miRNA genes. Seventeen of these new miRNAs (13 captured only by the HD adapters) could not have been found previously as they are not included in any of the raw sequences deposited in miRBase from more than 100 different deep sequencing experiments. It is therefore reasonable to suggest that new miRNAs will be identified in other tissues, especially in brain tissue, which shows the most diverse miRNA population.

Another consequence of the ligation bias is the potential mis-annotation of the two strands of a miRNA duplex. The active 'mature miRNA' is usually determined by higher read numbers compared to the 'star' sequence and these frequencies can be estimated by the ratio of counts of the two strands. However, these estimates are also prone to be distorted by ligation biases potentially leading to incorrect annotation of mature and star. We compared the count ratios for all annotated pairs of miRNAs derived from the same precursor expressed at a moderate to high level (>10 reads per million), using the DLD-1 Illumina and HD data sets. Although the correlation between the ratios obtained with the two protocols was relatively strong (R^2^ = 0.69, data not shown), we found 15 pairs out of the analysed 122 miRNA/miRNA* pairs for which the miRNA strand with a higher read number was different in the data obtained with Illumina and HD adapters (Additional file 10: Table S4).

### Bias Is Observed in MiRBase

Illumina and 454 have been the dominant technologies used for sRNA discovery (Additional file 11: Figure S6). We asked whether the miRNA research community had been biased by the dominant use of Illumina and 454 NGS, by analysis of data in miRBase, the global repository for miRNA data [33]. We found that miRNAs that were discovered with Illumina platforms were predicted to fold more strongly (more negative MFE) with Illumina adapters (both sRNA cloning kit V1.0 and V1.5 3' adapters) but less well with 454 adapters. The converse was also true; miRNAs that were discovered with the 454 technology were predicted to fold more strongly with 454 adapters but less well with Illumina adapters (Figure 3e-f). This is particularly unexpected because we did not take read number into account; i.e. if a miRNA was sequenced at least once by Illumina or 454 it was counted for the given platform. The majority of miRNAs in miRBase have been discovered using the Illumina platform; therefore the entire miRNA field became biased towards miRNAs that were preferred by the Illumina adapters.

## Discussion

The dominant use of Illumina technology has potentially biased the focus of the research community because the highly inaccurate quantification of miRNAs by the Illumina adapters could lead researchers to miss some interesting miRNAs. We present an approach to prepare substantially less biased sRNA libraries using HD adapters. Although in most cases the Illumina and HD adapters gave qualitatively similar results, the HD adapters dramatically improved measurement for some miRNAs. The most significant improvement over the Illumina protocol is the more accurate quantification of miRNA levels based on read numbers (Additional file 6: Table S2 and Additional file 7: Figure S4). For example, miR-29b is shown by quantitative Northern blot and HD sequencing to be the most abundant miRNA in DLD-1 cells. However, read counts from Illumina data rank miR-29b as only the 29th most abundant miRNA. Based on the Illumina result, it is highly unlikely that miR-29b would be chosen for a detailed analysis but the profile obtained by HD adapters could prompt further studies on miR-29b. Therefore ligation bias has probably led to inappropriate prioritisation of miRNAs for expensive follow-up experimental work. The bias is the same in all samples; therefore it could be argued that the bias is not important when two or more samples are compared and differentially expressed miRNAs are identified. However, read counts are taken into consideration not just when the miRNA content of one sample is studied, but when two or more samples are compared. It is more likely that a differentially expressed miRNA with high read number in one of the samples is chosen for functional studies than a differentially expressed miRNA with low read numbers in all samples. Although conservation and other factors also influence which miRNAs are chosen for further analysis, highly expressed miRNAs are often given priority (Figure 3d).

It is currently not understood if the highly expressed miRNAs are more active or if these miRNAs have many or highly expressed targets. Therefore quantitative measurement using HD adapters will help elucidate the relationship between miRNA and target gene expression levels. The HD approach is somewhat similar to the digital sequencing protocols, which apply individual barcodes to each cDNA molecule in the starting library and at the end the number of individual barcodes are counted instead of the number of total reads for each cDNA [37]. In principle, the number of degenerated nucleotides can be optimised in the future to accommodate a similar approach for counting small RNA copy numbers.

In a limited number of cases the HD adapters revealed a different ratio for the 5p and 3p strands of miRNAs than the Illumina adapters. The more abundant mature miRNA is often assumed to be the functional sequence, and is annotated as such. The ratio of the two strands can change in different tissues or during development. Since HD adapters are more quantitatively accurate, the annotation of the two strands should be more precise using the new adapters. Indeed we found that the two strands of 12.2% of the conserved miRNAs present in the DLD-1 cell line would be annotated differently based on the Illumina and HD data. This suggests that in any one experiment that uses the Illumina adapters, 10-15% of miRNAs may have the mature/star strands mis-annotated.

The sequence preferences from the two independent batches of degenerate libraries appeared unusually enhanced compared to the calculated probability of cloning a sequence. In optimal conditions Hafner et al. [21] found that the average ligation efficiency in a pool of RNAs was around 21%; therefore if the ligases were completely biased we would expect the complexity of the N21 cDNA library to be extremely large (~2 × 10^11^ sequences). Since we only sequenced 2 × 10^7^ sequences, we would have expected to observe each sequence read once if ligation conditions were optimal. In biological samples, the adapters are in excess to the small RNAs; however we used high amounts of degenerate oligonucleotides to ensure all possible sequences are represented. Therefore the limiting amount of adapters used would accentuate the sequence preferences of the ligase observed in the degenerate libraries.

We demonstrate that sequencing cDNA libraries generated by RNA ligases by NGS is an effective approach to study preferences of RNA ligases. A better understanding of Rnl2 function will allow the design of more efficient cloning protocols, such as HD adapters. This analysis may also shed light on the in vivo function of Rnl2, which is currently unknown. We note that our data are consistent with the proposed in vivo function of the related Rnl2 editing complex of trypanosome since the complex has been shown to prefer single-stranded residues at the ligation site [38]. NGS has transformed the way DNA/RNA sequence data are collected. Here we show that it can also be used to characterise enzyme specificities. We envisage that this approach could be modified to study many other nucleic acid modifying proteins.

## Materials and methods

### Functional Analysis of Nucleic Acid Modifying Enzymes Using NGS

This approach uses synthesised libraries containing millions of different possible nucleotide sequences that act as substrates for the enzyme of interest. We designed completely degenerate oligonucleotides such that each individual sequence is close to equimolar, which we call degenerate libraries. We add the enzyme of interest and subject the resulting sample to next-generation sequencing (Figure 1a).

### Quantitative Northern Blotting

For quantification of Northern blot analysis a calibration curve was generated. DNA oligonucleotides were quantified by nanodrop and serially diluted. Between 1.0, 2.5, 5.0, 7.5, 10.0, 25.0 and 50.0 nmols were loaded in an individual lane of a 15% denaturing polyacrylamide gel. Then 10 ug total cellular RNA was separated on a denaturing 15% polyacrylamide gel and transferred onto a nylon membrane as previously described [39]. Antisense DNA oligonucleotide probes were labelled with [gamma-^32^P]-ATP using PNK and detected using phosphorimager screens (Fujifilm). The Biorad molecular imager, FX pro plus, was used for signal visualisation, and ImageJ software was used for quantification of signal strength and image processing.

### Cell Lines and Cell Culture

DLD-1 wild-type and DLD-1 dicer−/− exon5 deletion were purchased from Horizon Discovery (Cambridge, UK). The colon epithelial adenocarcinoma DLD-1 cell lines were cultured in DMEM/F-12 + Glutamax (Gibco, 31331), supplemented with 10% FBS (PAA, A15-101) and 2% penicillin-streptomycin (Gibco, 15140). Cells were passaged using 0.25% Trypsin-EDTA (Gibco). Cells were grown in a 37°C, 5% CO_2_ humidified incubator.

### RNA Ligase Assays

In vitro assays of ligation activity were performed using substrates as described previously [40]. Oligonucleotides are listed in Additional file 12: Table S5.

### Small RNA Library Preparation

The N9 and N21 RNA oligonucleotides were chemically synthesised by Dharmacon. The nucleotide monomers were mixed in proportions to account for the differing coupling efficiencies of each monomer, and according to Dharmacon's description the difference between the incorporation of the four bases is expected to be less than 5%. For N9 RNA and N21 RNA cloning for NGS approximately 3 and 100 pmoles of oligonucleotide were used respectively. For biological samples, total RNA was isolated from DLD-1 or DLD-1 dicer−/− exon5 deletion using Trizol extraction buffer (Invitrogen). The small RNAs were enriched from at least 10 ug of total RNA using the *mir*VANA miRNA isolation Kit (Ambion). Library preparation was based on the Illumina small RNA v1.5 sample preparation guide. Approximately 200 ng of a small RNA-enriched sample was ligated to the pre-adenylated 3' adapter (custom synthesised by Bioo Scientific) with T4 Rnl2 truncated ligase (NEB). The ligated fragment was then ligated to the 5' adapter (Dharmacon) using T4 Rnl1. The ligated fragment was then reverse transcribed using the SRA RT primer followed by 8–14 cycles of PCR. The PCR products were size fractionated by polyacrylamide gel electrophoresis (8% PAGE). A band corresponding to approximately 100 bp was gel purified and sent for NGS sequencing on an Illumina Genome Analyzer IIx with 50 nt read length (Baseclear). Sequencing was performed in duplicate.

### Read Count Distributions

Because the sequencing procedure is essentially a sampling process where the sample is very large (>10^7^) and the frequencies are very low, under the assumption of equimolarity, the observed number of counts should be well approximated by a Poisson distribution [41]. For each library the parameter λ of the distribution is equal to n/p, where n is the total number of sequences in the sample and p is the total number of molecules contained in the library: for the N21 libraries p = 4^21^, for the N9 libraries p = 4^9^. To test the equimolarity hypothesis we compared the theoretical Poisson count distribution with the observed count distributions using a *χ*^2^-test.

### New Mirnas and RNA Secondary Structure

DLD sequencing data sets were processed with miRCat [22], using default parameters. The list of candidates was filtered based on fold change relative to Dicer-KO samples (> 1.4) and on the detection of a star sequence in data sets that had been integrated into miRBase [33].

All secondary structure predictions were obtained using RNAfold [42]. Temperature was set to 22°C; all other parameters were left at their default values.

### Mirbase Analyses

Using the annotation in miRBase (version 17) we retrieved the NGS platforms with which each miRNA has been detected. This information was used to split the set of miRNAs as shown in Additional file 11: Figure S6. The same split was used to create Figure 3e-f and Additional file 5: Figure S7.

## Abbreviations

Ds: Double stranded; HD: High definition; HiTS-FLIP: High-throughput sequencing-fluorescent ligand interaction profiling; MFE: Minimum free energy; miRNA: MicroRNA; NGS: Next generation sequencing; qPCR: Quantitative PCR; Rnl: RNA ligase; sRNA: Small RNA; ss: Single stranded.

## Competing interests

The authors declare no competing financial interests.

## Authors’ contributions

The project was conceived and experiments planned by K.S., H.P. and T.D, and K.S. carried out experiments. A.E.H. cultured all cell lines used in the paper, extracted RNA and prepared the Northern blots. H.P., A.K. and S. G.-J. performed data analysis. K.S., H. P and T.D. wrote the manuscript, and all authors reviewed it. The data analysis aspects of the study were supervised by V.M. and all aspects of the study were supervised by T.D. All authors read and approved the final manuscript

## Supplementary Material

Additional file 1 **Table S1.** Number of reads of the 10,000 most abundant sequences for N21 RNA library prepared with Illumina adapters. Most abundant sequences and their read numbers in the N21 library generated with Illumina adapters. Click here for file

Additional file 2 **Figure S1.** Number of reads for the 100 most abundant sequences in the N9 libraries, prepared with Illumina (red) or HD adapters (blue). Click here for file

Additional file 3 **Figure S2.** Frequencies of predicted nucleotide base-pairing per position for N21 insert and 3’ HD adapter (a) and 5’ HD adapter, insert and 3’ HD adapter (b). Vertical dotted line indicates ligation point. Blue line denotes data obtained with HD protocol and grey line randomly generated sets of 21nt sequences. Bars indicate minimum and maximum values in all replicates. Click here for file

Additional file 4 **Figure S3.** Number of barcode pairs that capture sequences in N9 library prepared with HD adapters. The majority of sequences are captured by a number of barcodes much smaller than the total number of barcode combinations (65,536). Click here for file

Additional file 5 **Figure S7.** miRNAs in miRBase show bias towards 454 and Illumina adapters. Distributions of minimum free energy (MFE) of known human miRNAs concatenated only with 3’ adapter sequences. Using Illumina adapter sequences the set of miRNAs found by Illumina has lower average MFE than the set of miRNAs found by 454 (left). Conversely, using 454 adapter sequences average MFE is lower for set of miRNAs found by 454 (right). Click here for file

Additional file 6 **Table S2.** Most abundant miRNAs in DLD-1 cells obtained with Illumina or HD adapters. Sequences, read numbers, miRNA names, normalised read numbers (read per million) and rank based on normalised read number are shown for the most abundant miRNAs in DLD-1 cells in libraries generated with either Illumina or HD adapters. Click here for file

Additional file 7 **Figure S4.** Validation of miRNA read numbers by quantitative Northern blot. Quantification of known microRNAs in DLD-1 cells (epithelial adenocarcinoma cells), both wild type and dicer−/−. Total RNA (10 μg) was analysed via Northern blot and microRNA experimental samples were quantified using a serial dilution of synthetic oligonucleotide sequences corresponding with the miR of interest. The small nuclear RNA U6 was used as a loading control, and this U6 image has been duplicated where different microRNAs were analysed on the same membrane (miRs 10a & 29b and miRs 25 & Let 7i). Click here for file

Additional file 8 **Table S3.** New miRNAs identified in DLD-1 cells. Sequences and various characteristics of new miRNAs identified by miRCat in DLD-1 cells. Read numbers (in million reads) are shown for each library (dlddc1il: DLD-1 Dicer KO repeat 1 with Illumina adapters; dlddc2il: DLD-1 Dicer KO repeat 2 with Illumina adapters; dldwt1il: DLD-1 repeat 1 with Illumina adapters; dldwt2il: DLD-1 repeat 2 with Illumina adapters; dlddc1hd: DLD-1 Dicer KO repeat 1 with HD adapters; dlddc2hd: DLD-1 Dicer KO repeat 2 with HD adapters; dldwt1hd: DLD-1 repeat 1 with HD adapters; dldwt2hd: DLD-1 repeat 2 with HD adapters). The last two columns indicate which new miRNAs were found with which adapters. Click here for file

Additional file 9 **Figure S5.** Secondary structures of new miRNAs. The secondary structure of each new miRNA is shown in bracket notation and also where all the sequencing reads map on the pre-miRNA. The two numbers on the right-hand side are the sums of counts for the two HD wild-type replicates (left) and for the two Illumina wild-type replicates (right). Click here for file

Additional file 10 **Table S4.** Identification of the more abundant miRNA strand MiRNAs were selected with normalised read number >10 per million reads in either the Illumina or HD library. Read numbers are shown for both strands of the miRNA duplex in the wild-type DLD-1 libraries obtained either with the Illumina or the HD adapters (dldwt1il: DLD-1 repeat 1 with Illumina adapters; dldwt2il: DLD-1 repeat 2 with Illumina adapters; dldwt1hd: DLD-1 repeat 1 with HD adapters; dldwt2hd: DLD-1 repeat 2 with HD adapters) in columns d-G. Columns I and J show the read cumulative numbers of the replicates (wtil: DLD-1 with Illumina adapters; wthd: DLD-1 with HD adapters). Column K shows the current name of each miRNA strand (5p, 3p, mature or star). Columns L and M show thelog2 of the ratio wt/dld (HD and Illumina, respectively). Column N shows the direction of the change between Illumina and HD, i.e. if it is −1 there is an arm switch. Click here for file

Additional file 11 **Figure S6.** Numbers of miRNAs in miRBase sequenced by each next generation sequencing platform. Most miRNAs were discovered with Illumina and 454 technology. Proportion of miRNAs that were discovered with the main NGS technologies as identified in miRBase. Click here for file

Additional file 12 **Table S5.** Oligonucleotide sequences used in during the study. List of oligonucleotides in 5' to 3' orientation. n = denegenerate nucleotide. r = RNA. Fl = Fluoroscein. Phos = Phosphorylated. rApp = adenylated. 3ddC and sAaMO = 3' blocking group. AmMC6 = 5' blocking group. Red shows regions of interest and capitals highlight nucleotide changes compared to control template. Click here for file
