# Supplementary material for: Reducing ligation bias of small RNAs in libraries for next generation sequencing
Source: Silence. 2012 May 30;3:4. doi: 10.1186/1758-907X-3-4 (PMC3489589; doi:10.1186/1758-907X-3-4)
Supplement: Additional file 2 — Figure S1. Number of reads for the 100 most abundant sequences in the N9 libraries, prepared with Illumina (red) or HD adapters (blue). [file 1758-907X-3-4-S2.pdf]

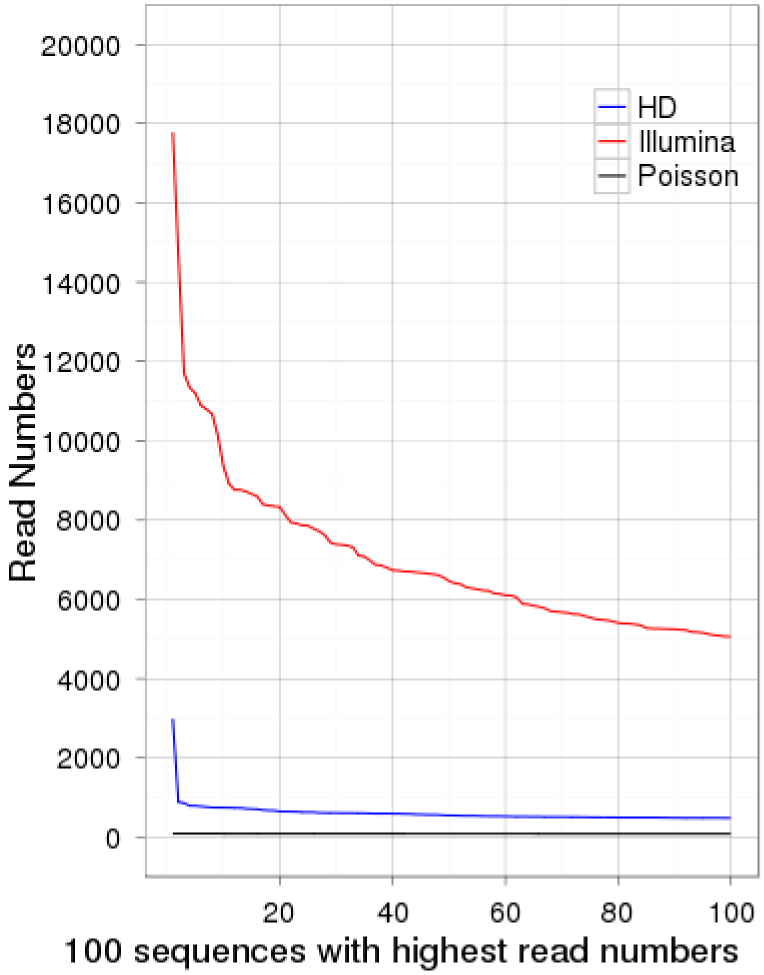

Supplementary figure 1. Number of reads for the 100 most abundant sequences in the N9 libraries, prepared with Illumina (red) or HD adapters (blue).
