# Supplementary material for: Reducing ligation bias of small RNAs in libraries for next generation sequencing
Source: Silence. 2012 May 30;3:4. doi: 10.1186/1758-907X-3-4 (PMC3489589; doi:10.1186/1758-907X-3-4)
Supplement: Additional file 3 — Figure S2. Frequencies of predicted nucleotide base-pairing per position for N21 insert and 3’ HD adapter (a) and 5’ HD adapter, insert and 3’ HD adapter (b). Vertical dotted line indicates ligation point. Blue line denotes data obtained with HD protocol and grey line randomly generated sets of 21nt sequences. Bars indicate minimum and maximum values in all replicates. [file 1758-907X-3-4-S3.pdf]

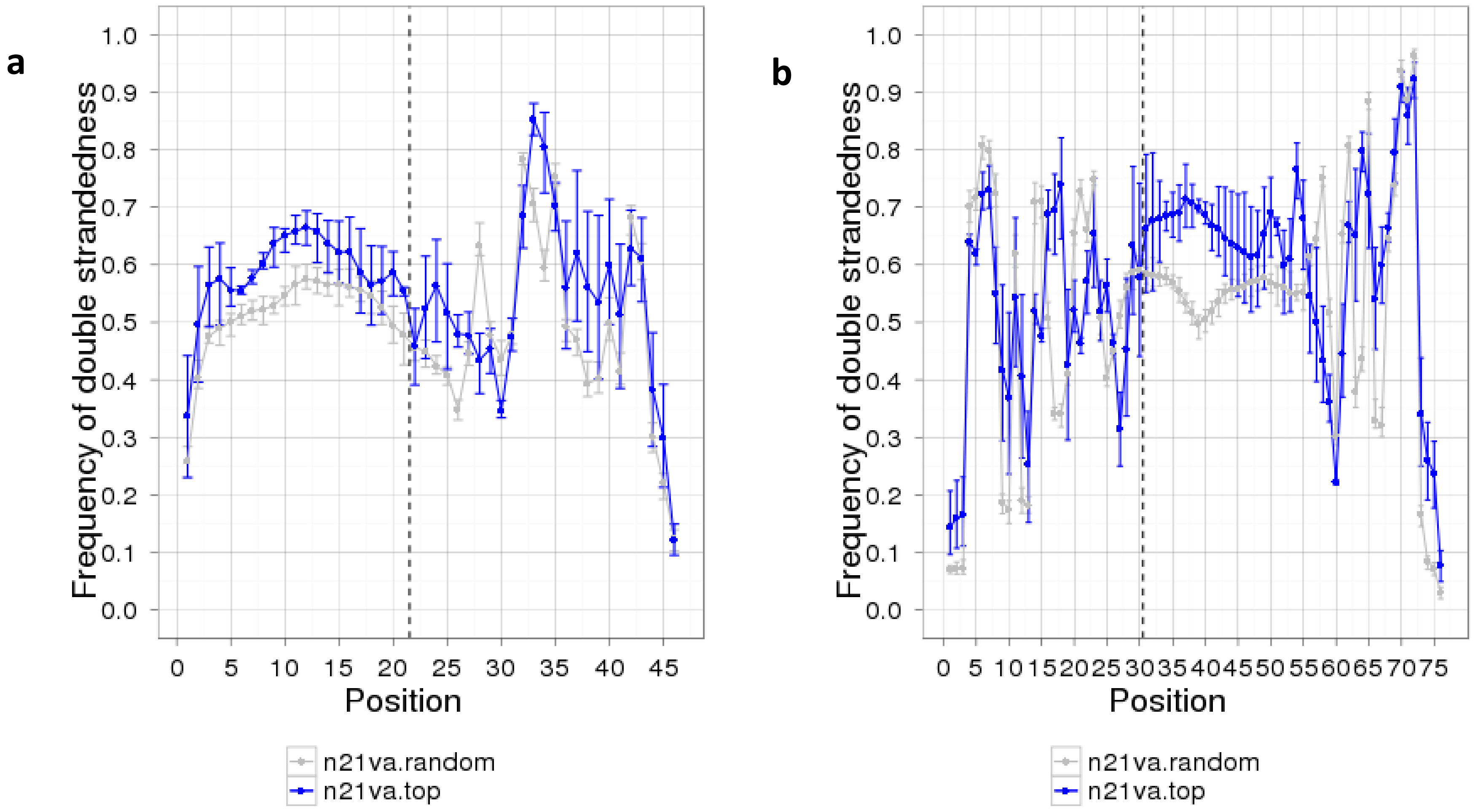

Supplementary Figure 2. Frequencies of predicted nucleotide base-pairing per position for N21 insert and 3’ HD adapter (a) and 5’ HD adapter, insert and 3’ HD adapter (b). Vertical dotted line indicates ligation point. Blue line denotes data obtained with HD protocol and grey line randomly generated sets of 21nt sequences. Bars indicate minimum and maximum values in all replicates.
