# Supplementary material for: Reducing ligation bias of small RNAs in libraries for next generation sequencing
Source: Silence. 2012 May 30;3:4. doi: 10.1186/1758-907X-3-4 (PMC3489589; doi:10.1186/1758-907X-3-4)
Supplement: Additional file 4 — Figure S3. Number of barcode pairs that capture sequences in N9 library prepared with HD adapters. The majority of sequences are captured by a number of barcodes much smaller than the total number of barcode combinations (65,536). [file 1758-907X-3-4-S4.pdf]

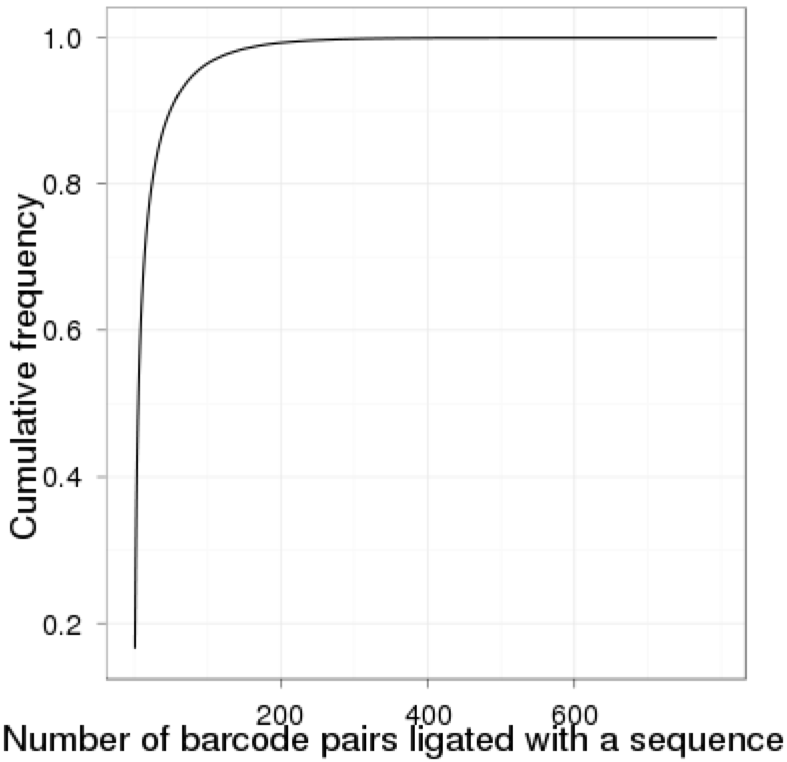

Supplementary figure 3. Number of barcode pairs that capture sequences in N9 library prepared with HD adapters. The majority of sequences are captured by a number of barcodes much smaller than the total number of barcode combinations (65,536).
