# Supplementary material for: Reducing ligation bias of small RNAs in libraries for next generation sequencing
Source: Silence. 2012 May 30;3:4. doi: 10.1186/1758-907X-3-4 (PMC3489589; doi:10.1186/1758-907X-3-4)
Supplement: Additional file 5 — Figure S7. miRNAs in miRBase show bias towards 454 and Illumina adapters. Distributions of minimum free energy (MFE) of known human miRNAs concatenated only with 3’ adapter sequences. Using Illumina adapter sequences the set of miRNAs found by Illumina has lower average MFE than the set of miRNAs found by 454 (left). Conversely, using 454 adapter sequences average MFE is lower for set of miRNAs found by 454 (right). [file 1758-907X-3-4-S5.pdf]

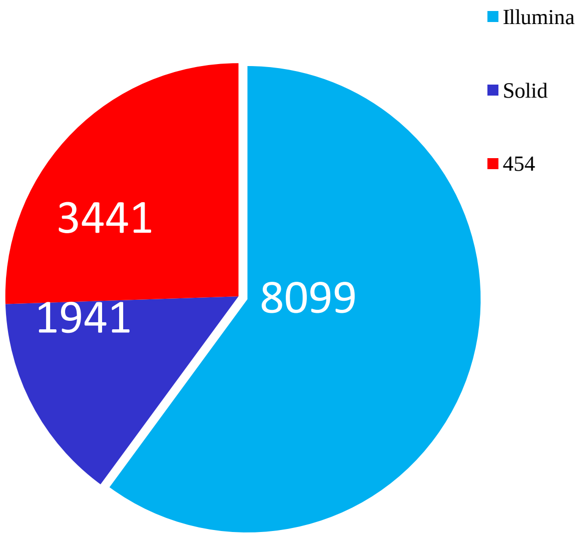

Supplementary figure 6. Most miRNAs were discovered with Illumina and 454 technology. Proportion of miRNAs that were discovered with the main NGS technologies as identified in miRBase.
