# Supplementary material for: Reducing ligation bias of small RNAs in libraries for next generation sequencing
Source: Silence. 2012 May 30;3:4. doi: 10.1186/1758-907X-3-4 (PMC3489589; doi:10.1186/1758-907X-3-4)
Supplement: Additional file 7 — Figure S4. Validation of miRNA read numbers by quantitative Northern blot. Quantification of known microRNAs in DLD-1 cells (epithelial adenocarcinoma cells), both wild type and dicer−/−. Total RNA (10 μg) was analysed via Northern blot and microRNA experimental samples were quantified using a serial dilution of synthetic oligonucleotide sequences corresponding with the miR of interest. The small nuclear RNA U6 was used as a loading control, and this U6 image has been duplicated where different microRNAs were analysed on the same membrane (miRs 10a & 29b and miRs 25 & Let 7i). [file 1758-907X-3-4-S7.pdf]

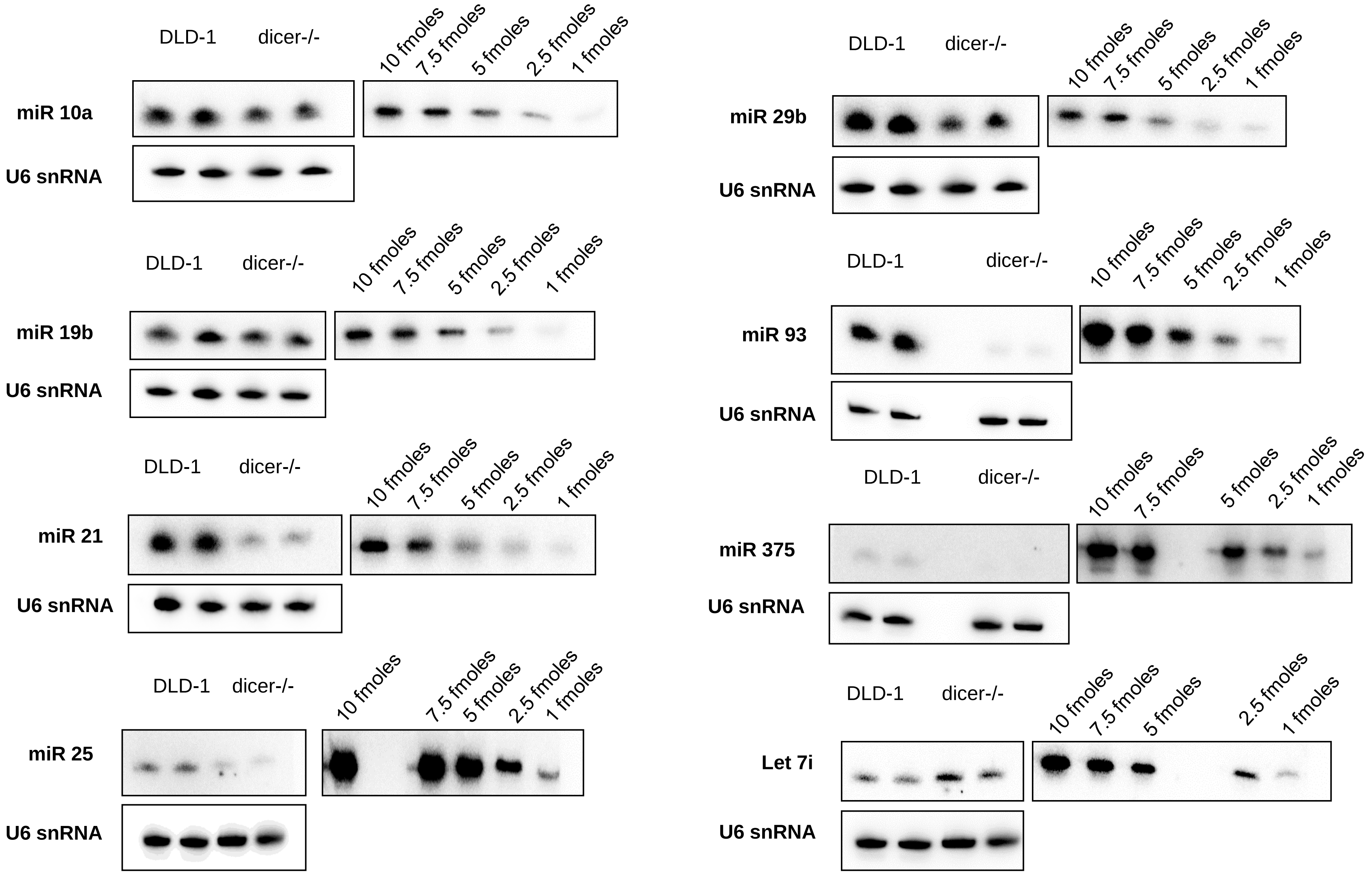

Supplementary Figure 4. Quantification of known microRNAs in DLD-1 cells (epithelial adenocarcinoma cells), both wild-type and dicer-/-. Total RNA (10µg) was analysed via Northern blot and microRNA experimental samples were quantified using a serial dilution of synthetic oligonucleotide sequences corresponding with the miR of interest. The small nuclear RNA U6 was used as a loading control, and this U6 image has been duplicated where different microRNAs were analysed on the same membrane (miRs 10a & 29b and miRs 25 & Let 7i).
