# Supplementary material for: Reducing ligation bias of small RNAs in libraries for next generation sequencing
Source: Silence. 2012 May 30;3:4. doi: 10.1186/1758-907X-3-4 (PMC3489589; doi:10.1186/1758-907X-3-4)
Supplement: Additional file 10 — Table S4. Identification of the more abundant miRNA strand MiRNAs were selected with normalised read number >10 per million reads in either the Illumina or HD library. Read numbers are shown for both strands of the miRNA duplex in the wild-type DLD-1 libraries obtained either with the Illumina or the HD adapters (dldwt1il: DLD-1 repeat 1 with Illumina adapters; dldwt2il: DLD-1 repeat 2 with Illumina adapters; dldwt1hd: DLD-1 repeat 1 with HD adapters; dldwt2hd: DLD-1 repeat 2 with HD adapters) in columns d-G. Columns I and J show the read cumulative numbers of the replicates (wtil: DLD-1 with Illumina adapters; wthd: DLD-1 with HD adapters). Column K shows the current name of each miRNA strand (5p, 3p, mature or star). Columns L and M show thelog2 of the ratio wt/dld (HD and Illumina, respectively). Column N shows the direction of the change between Illumina and HD, i.e. if it is −1 there is an arm switch. [file 1758-907X-3-4-S10.pdf]

Supplementary Figure 5.

The secondary structure of each new miRNA is shown in bracket notation and also where all the sequencing reads map on the pre-miRNA. The two numbers on the right-hand side are the sums of counts for the two HD wild type replicates (left) and for the two Illumina wild type replicates (right).

```
chr17 40646791-40646812

 TTAGGTTTGGTGCAAAAGTTATTGCGGTTTTGGCTGCCAAAAGAAATGGCCAAAACCGCAGTAACTTTTGTGCCAACCTAA

 (((((((.((..(<<<<<<<<<<<<<<<<<<<<<<............)))))))))))))))))))))))..)))))))))

              AAAAGTTATTGCGGTTTTGGCT                                                  8,  1

              AAAAGTTATTGCGGTTTTGGC                                                   1,  0

              AAAAGTTATTGCGGTTTTGG                                                    1,  1


chr1  219563096-219563117

 TATATTAGGTTGGCGTGAAAGTAATTTCGGTTTTTGCCATTACTTTTAGTGGCAAAAATCGCAATTACTTTTGTGCCAACCTAATATA

 ((((((((((((((..(<<<<<<<<<-<<<<<<<<<<<<(((....))))))))))))))).)))))))))..)))))))))))))))

                  AAAGTAATTTCGGTTTTTGCCA                                                     2,  0

                  AAAGTAATTTCGGTTTTTGCCAT                                                    1,  0

                  AAAGTAATTTCGGTTTTTGC                                                       1,  3

                  AAAGTAATTTCGGTTTTTGCC                                                      1,  0

                   AAGTAATTTCGGTTTTTGCCAT                                                    8,  1

                   AAGTAATTTCGGTTTTTGCCA                                                     5,  2
```

```
                   AAGTAATTTCGGTTTTTGCCATT                                                      4,  0
                    AGTAATTTCGGTTTTTGCCATT                                                      3,  0
                                                     CAAAAATCGCAATTACTTTTG                      0,  2


chr3  129618465-129618486
 AGGTTGGTGCAAAAGTAATTTCGGTTTTTGCCATTACTCACTGCATTACTTTGGCAAAAACCAAAATTACTTTTGCACCAACCT
 ((((((((((<<<<<<<<<<<-<<<<<<<<<((..................))))))))))).)))))))))))))))))))))
          CAAAAGTAATTTCGGTTTTTGCC                                                          1,  2
          CAAAAGTAATTTCGGTTTTTG                                                            1,  0
           AAAAGTAATTTCGGTTTTTGC                                                           5,  0
           AAAAGTAATTTCGGTTTTTGCC                                                          3,  1
            AAAGTAATTTCGGTTTTTGCCA                                                         2,  0
            AAAGTAATTTCGGTTTTTGCC                                                          1,  0
            AAAGTAATTTCGGTTTTTGCCAT                                                        1,  0
            AAAGTAATTTCGGTTTTTGC                                                           1,  3
             AAGTAATTTCGGTTTTTGCCAT                                                        8,  1
             AAGTAATTTCGGTTTTTGCCA                                                         5,  2
             AAGTAATTTCGGTTTTTGCCATT                                                       4,  0
              AGTAATTTCGGTTTTTGCCATT                                                       3,  0
                                                       CAAAAACCAAAATTACTTTTGC              1,  0
```

chr5 161136476-161136497

```
 AGTATTAATTGATGGTGCAAAAGTAATTTCGGTTTTTGCCATTATTTTTAATGGCAAAAAACCGCAATTATTTTTGCACCAATCTAATATT

 (((((((...((((((((((<<<<<<<<-<<<<<<<<<<<<(((....))))))).)))))))).))))))))))))))).))))))))))

                  CAAAAGTAATTTCGGTTTTTGCC                                                       1,  2

                  CAAAAGTAATTTCGGTTTTTG                                                         1,  0

                   AAAAGTAATTTCGGTTTTTGC                                                        5,  0

                   AAAAGTAATTTCGGTTTTTGCC                                                       3,  1

                    AAAGTAATTTCGGTTTTTGCCA                                                      2,  0

                    AAAGTAATTTCGGTTTTTGCCAT                                                     1,  0

                    AAAGTAATTTCGGTTTTTGCC                                                       1,  0

                    AAAGTAATTTCGGTTTTTGC                                                        1,  3

                     AAGTAATTTCGGTTTTTGCCAT                                                     8,  1

                     AAGTAATTTCGGTTTTTGCCA                                                      5,  2

                     AAGTAATTTCGGTTTTTGCCATT                                                    4,  0

                      AGTAATTTCGGTTTTTGCCATT                                                    3,  0

                                                         AAAAACCGCAATTATTTTTGC                  6,  0

                                                          AAAACCGCAATTATTTTTGC                  2,  0
```

```
chr15 90393919-90393942

 GAAAAAAATCAGTGAATGCCTTGAACCTAACACACTGCCTTTTATGTGGTAGGTACAGTGGGCTCACTGAAACATTC

 (((....-<<<<<<<--<<<<<<-<<<<<-<(((..........)))).))))).))..)))))))))))....)))

       AATCAGTGAATGCCTTGAACCTAAC                                                  1,  17

       AATCAGTGAATGCCTTGAACCTA                                                    0,  3

       AATCAGTGAATGCCTTGAACC                                                      0,  2

       AATCAGTGAATGCCTTGAACCT                                                     0,  1

        ATCAGTGAATGCCTTGAACCTAAC                                                  7,  50

        ATCAGTGAATGCCTTGAACCTA                                                    5,  2

        ATCAGTGAATGCCTTGAACCTAA                                                   1,  0

        ATCAGTGAATGCCTTGAACC                                                      0,  1

        ATCAGTGAATGCCTTGAACCTAACAC                                                0,  1

         TCAGTGAATGCCTTGAACCTAAC                                                  2,  9

         TCAGTGAATGCCTTGAACCTAACA                                                 2,  2

                                                 GTAGGTACAGTGGGCTCACTGAAA         0,  3

                                                 GTAGGTACAGTGGGCTCACTGAA          0,  1

                                                 GTAGGTACAGTGGGCTCACTGA           0,  1

                                                  TAGGTACAGTGGGCTCACTGA           1,  1

                                                  TAGGTACAGTGGGCTCACTGAAAC        0,  2

                                                  TAGGTACAGTGGGCTCACTGAAA         0,  3
```

chr1 214673356-214673377

CATCATCTAGAATGAGAAAATGTCCCACATTTGTGGACATTTGCGGACAATGTGTGACATTTTCTCATTCTAGATGATG

(((((((((({{{{{{{{{{{{{{={{{{{{{(..(....)..))..->>>>>>->>>>>>>>>>>>>>))))))))))

AATGAGAAAATGTCCCACATTT 10, 0

AATGAGAAAATGTCCCACATT 3, 0

ATGAGAAAATGTCCCACATTTGT 1, 0

TGAGAAAATGTCCCACATTTGT 4, 0

CAATGTGTGACATTTTCTCATT 14, 0

CAATGTGTGACATTTTCTCAT 1, 0

AATGTGTGACATTTTCTCATTC 2, 0

ATGTGTGACATTTTCTCATTCT 23, 0

ATGTGTGACATTTTCTCATTC 1, 0

TGTGTGACATTTTCTCATTCT 8, 1

TGTGTGACATTTTCTCATTC 1, 0

chr7 134891796-134891816

TTTTTGTGTGTGAAATTAGGTAGTGGCAGTGGAACACTATATTAATCAGGTTTCCACTGCCACTACCTAATTTCTCAGATGGAAA

((((..(.((.(((<<<<<<<<<<<<<<<<<<<<-<((..........)))))))))))))))))))))))))).)).)..))))

ATTAGGTAGTGGCAGTGGAACA 5, 42

ATTAGGTAGTGGCAGTGGAACA	5, 42
ATTAGGTAGTGGCAGTGGAACA	5, 42
ATTAGGTAGTGGCAGTGGAACA	5, 42
ATTAGGTAGTGGCAGTGGAAC	1, 23
ATTAGGTAGTGGCAGTGGAAC	1, 23
ATTAGGTAGTGGCAGTGGAAC	1, 23
ATTAGGTAGTGGCAGTGGAAC	1, 23
ATTAGGTAGTGGCAGTGGAACAC	0, 2
ATTAGGTAGTGGCAGTGGAACAC	0, 2
ATTAGGTAGTGGCAGTGGAACAC	0, 2
ATTAGGTAGTGGCAGTGGAACAC	0, 2
ATTAGGTAGTGGCAGTGGAA	0, 5
ATTAGGTAGTGGCAGTGGAA	0, 5
ATTAGGTAGTGGCAGTGGAA	0, 5
ATTAGGTAGTGGCAGTGGAA	0, 5
TTAGGTAGTGGCAGTGGAACA	0, 5
TTAGGTAGTGGCAGTGGAACA	0, 5
TTAGGTAGTGGCAGTGGAACAC	0, 1
TTAGGTAGTGGCAGTGGAACAC	0, 1
TTAGGTAGTGGCAGTGGAACA	0, 5

```
        TTAGGTAGTGGCAGTGGAACA                                                   0,  5
        TTAGGTAGTGGCAGTGGAACAC                                                  0,  1
        TTAGGTAGTGGCAGTGGAACAC                                                  0,  1

chr3  46969938-46969959
AGGTTGGTGCAAAAGTAATTGTGGTTTCTGCCACTATGTTCAATGGCAAAAACCACAGTTATTTTTGCATCAACCT
(((((((((({{{{{{{{{{{{{{{{{={{{{(..........)))>>->>>>>>>>>>>>>>>>>>))))))))
   TTGGTGCAAAAGTAATTGTGGTT                                              0,  1
    TGGTGCAAAAGTAATTGTGGTT                                              0,  2
     GGTGCAAAAGTAATTGTGGTTT                                             1,  0
     GGTGCAAAAGTAATTGTGGTT                                              0,  1
          AAAAGTAATTGTGGTTTCTGC                                         3,  1
          AAAAGTAATTGTGGTTTCTGCC                                        2,  3
          AAAAGTAATTGTGGTTTCTGCCA                                       0,  1
          AAAAGTAATTGTGGTTTCTG                                          0,  1
           AAAGTAATTGTGGTTTCTGCCA                                       0,  1
                                         TGGCAAAAACCACAGTTATTTT         1,  0
                                            CAAAAACCACAGTTATTTTTGC      9,  0
                                             AAAAACCACAGTTATTTTTGCA     1,  0
```

```
chr9  37296068-37296089

 TTTAGGTTGGTGCAAAAGTAGTTGTGGTTTTTGCCATTACTTTCAAAGGCATAAACCACAATTACTTTTGCACCAGCCTAAA

 ((((((((((((({{{{{{{{{{{{{{{{{={{{{............))>>->>>>>>>>>>>>>>>>>>>)))))))))))

             AAAAGTAGTTGTGGTTTTTGCC                                                5,  0

             AAAAGTAGTTGTGGTTTTTGC                                                 3,  0

             AAAAGTAGTTGTGGTTTTTG                                                  1,  0

                                                 CATAAACCACAATTACTTTTGC            11,  0

                                                    AAACCACAATTACTTTTGCACC         14,  10

                                                    AAACCACAATTACTTTTGCAC          7,  0

                                                     AACCACAATTACTTTTGCACCA        5,  1

                                                     AACCACAATTACTTTTGCACC         3,  1

                                                      ACCACAATTACTTTTGCACCA        25,  1

                                                      ACCACAATTACTTTTGCACC         5,  2


chr17 19834298-19834319

 CTGGGACTAGAGGCATGCACCACCACGCCTGGCTAATTTGAAATTTTTTTTTGGCCAGGCATGATGGTTCACGCCTGTAATCCCAG

 ((((((.((.((((.((.((((.((.((((((((((..............))))>>>>>>->>->>>>->>->>>>.)).))))))

          AGGCATGCACCACCACG                                                          1,  0

            GCATGCACCACCACGCCTGGCT                                                   2,  0

            GCATGCACCACCACGCCTGGC                                                    1,  1
```

```
              CATGCACCACCACGCCTGGCT                                                        1,  0
                                                       CCAGGCATGATGGTTCACGCCT              324,  2
                                                       CCAGGCATGATGGTTCACGCC               10,  0
                                                       CCAGGCATGATGGTTCACGCCTGT            1,  0
                                                       CCAGGCATGATGGTTCACG                 0,  1
                                                        CAGGCATGATGGTTCACGCCT              28,  0
                                                        CAGGCATGATGGTTCACGCC               9,  0
                                                        CAGGCATGATGGTTCACGCCTGT            1,  0
                                                         AGGCATGATGGTTCACGCCTGT            1,  0

chr2  150186814-150186835
 GCAAACTCCGGCCAGCGGGAGCCGACCGCCCGGCGCAGGTGGGGCACCCGGGCGCCGCGCTCCCGCCCGCAGGGTGAGC
 ((..((.((.((..((((((((((..(((((((.((.......))..)>>>>>>->>->>>>>>>>>--->>.)))))..))
        CCGGCCAGCGGGAGCCGACCGCCCGGCG                                                1,  0
         CGGCCAGCGGGAGCCGACCGCC                                                     0,  1
              AGCGGGAGCCGACCGCCCGG                                                  1,  1
                                                 CGGGCGCCGCGCTCCCGCCCGC             70,  1
                                                 CGGGCGCCGCGCTCCCGCCCG              18,  4
                                                 CGGGCGCCGCGCTCCCGCCCGCA            8,  0
                                                 CGGGCGCCGCGCTCCCGCCC               4,  2
```

CGGGCGCCGCGCTCCCGCC 1, 0

GGGCGCCGCGCTCCCGCCCGC 3, 0

GGGCGCCGCGCTCCCGCCCGCA 1, 0

chr1 160312313-160312334

```
CCAGTTAAATTTGAATTTCAGGTAAACAATGAATAGTTTTTAGTCTGTCGTTGCTTACCAGAAGTTCAAATTTATCTGG
((((.(((((((<<<<<<<<-<<<<<<-<<<<<<<-<((.........))}}}}}}}=}}}}}=}}}}}}}}))))))).))))
            GAATTTCAGGTAAACAATGAAT                                                     133,  1
            GAATTTCAGGTAAACAATGAA                                                      4,  0
            GAATTTCAGGTAAACAATGA                                                       3,  1
            GAATTTCAGGTAAACAATGAATA                                                    1,  0
             AATTTCAGGTAAACAATGAATA                                                    1,  0
             AATTTCAGGTAAACAATGAATAG                                                   1,  0
                                                 GTCGTTGCTTACCAGAAGTTC                 1,  0
                                                 GTCGTTGCTTACCAGAAGTTCA                1,  1
                                                   CGTTGCTTACCAGAAGTTCAA               3,  0
```

chr18 50403414-50403435

```
GTTAAATTTGAATTTCAGGTAAACAATGAATACATTTTTAGTATAAATGTGTACCATGCAATATTTTAGACACAATTATATTAAAAACATATTCATTGTTTATCTGAAATTCAAATTTAAC
(((((((((<<<<<<<<<<<<<<<<<<<<<<<(..((((((((((((.(((((................))))).))))))))))))..))))))))))))))))))))))))))))))))
```

```
GAATTTCAGGTAAACAATGAAT          133,  1
GAATTTCAGGTAAACAATGAA           4,  0
GAATTTCAGGTAAACAATGA            3,  1
GAATTTCAGGTAAACAATGAATA         1,  0
 AATTTCAGGTAAACAATGAATA         1,  0
```

chr12 50722573-50722593

```
TACAGGCATGAGCCACCACTCCTGGCCTCGAAGTAGGTTTTAAAACATGTCCAGGCCGGGCACGGTGGCTCACGCCTGTA
(((((((.(<<<<<<<<---<<<<<<<<-<(.....(((....)))...)).))))))))...))))))))).)))))))
         GAGCCACCACTCCTGGCCTCG                                                      10,  1
         GAGCCACCACTCCTGGCCTCGA                                                     1,  0
                                                  CAGGCCGGGCACGGTGGCTCACGCCTGTA     1,  0
                                                  CAGGCCGGGCACGGTGGCTCA             0,  1
                                                  CAGGCCGGGCACGGTGGCTCAC            0,  2
                                                    GGCCGGGCACGGTGGCTCACG           1,  0
                                                      CCGGGCACGGTGGCTCACGC          0,  2
                                                       CGGGCACGGTGGCTCACGCCTGT      1,  0
                                                       CGGGCACGGTGGCTCACGCCTG       1,  0
                                                          GCACGGTGGCTCACGCCTG       1,  0
```

chr8 30927639-30927660

TATTAGGTTGGTGCAAAAATAATTGTGGTTTTGGTAATTTTTAATGGCAAAAACCACAATTACTTTTGCACCAACCTAATA

(((((((((((<<<<<<<-<<<<<<<<<<<<<-((.((.....)).)).))))))))))))).))))))))))))))))))

GTGCAAAAATAATTGTGGTTT 9, 1

GTGCAAAAATAATTGTGGTT 1, 0

GTGCAAAAATAATTGTGGT 1, 0

GTGCAAAAATAATTGTGGTTTT 0, 1

TGCAAAAATAATTGTGGTTTTG 289, 29

TGCAAAAATAATTGTGGTTTT 12, 0

TGCAAAAATAATTGTGGTTTTGG 0, 4

GCAAAAATAATTGTGGTTTTGG 6, 23

GCAAAAATAATTGTGGTTTTG 3, 0

GCAAAAATAATTGTGG 0, 1

CAAAAATAATTGTGGTTTTGGT 1, 0

TGGCAAAAACCACAATTACTTTT 1, 0

GCAAAAACCACAATTACTTTTGC 6, 0

GCAAAAACCACAATTACTTTTG 4, 0

CAAAAACCACAATTACTTTTGC 41, 1

CAAAAACCACAATTACTTTTG 6, 1

AAAAACCACAATTACTTTTGCA 107, 2

```
                                AAAAACCACAATTACTTTTGC                86,  0
                                AAAAACCACAATTACTTTTGCAC              6,  0
                                AAAAACCACAATTACTTTTG                 3,  0
                                 AAAACCACAATTACTTTTGCAC              84,  1
                                 AAAACCACAATTACTTTTGCA               17,  1
                                 AAAACCACAATTACTTTTGC                3,  0
                                 AAAACCACAATTACTTTTG                 1,  0
                                 AAAACCACAATTACTTTTGCACC             1,  0
                                  AAACCACAATTACTTTTGCACC             14,  10
                                  AAACCACAATTACTTTTGCAC              7,  0
                                   AACCACAATTACTTTTGCACCA            5,  1
                                   AACCACAATTACTTTTGCACC             3,  1
                                    ACCACAATTACTTTTGCACCA            25,  1
                                    ACCACAATTACTTTTGCACC             5,  2
                                    ACCACAATTACTTTTGCACCAA           1,  0
                                          ATTACTTTTGCACCAACCTAAT     1,  0

chr2  152538493-152538515

 TATTAGGTTGGTACAAAAGTAATTGCAGTTTTGACATTAAAAGTAATGGCAAGAACCACAGTTACTTTTGCCCCAGCATAATA
 (((((.(((((..((((((((((((..(((((..(((((....))))))))))--->>>>>-->>>>>>>>>>>>-.))))).)))))
```

GCAAGAACCACAGTTACTTTTGC 16, 0

CAAGAACCACAGTTACTTTTGC 3, 3

chr18 19775466-19775487

TTAGGTTGGTGCAAAAGTAATTGTGGGTTTTGTCATTACTTTCAATGCCAAAAACTGCAATTACTTTGGCAACAACCTAA

((((((((.(((={{{{{{{{{{=={={{{{{={(((......)))>->>>>>->-->>>>>>>>>>-))).))))))))

TTAGGTTGGTGCAAAAGTAATTG 1, 1

CAAAAGTAATTGTGGGTTTTGTC 0, 1

AAAAGTAATTGTGGGTTTTGTC 4, 20

AAAAGTAATTGTGGGTTTTGT 0, 4

AAAAGTAATTGTGGGTTTTGTCA 0, 1

AAGTAATTGTGGGTTTTGTCAT 6, 11

AAGTAATTGTGGGTTTTGTC 2, 3

AAGTAATTGTGGGTTTTGTCA 1, 12

GCCAAAAACTGCAATTACTTTG 69, 27

GCCAAAAACTGCAATTACTTT 1, 0

CCAAAAACTGCAATTACTTTG 6, 11

CCAAAAACTGCAATTACTTTGG 1, 0

CAAAAACTGCAATTACTTTGGC 1, 0

CAAAAACTGCAATTACTTTGGCA 0, 1

chr1 1284690-1284711

GGCGCGGCCCGAGGGGCGCAGGCGGGAGGGCGGCGGGGCTGAGCGCGCCCTCCGTGCTGCGTCCCTCGCGCCGCTCC

((.(((((.((((((((((((.((((((((((((........)).)>>>>>>>->>>>>>>>>>>>>>>.))))).))

AGGGGCGCAGGCGGGAGGGCGGC	3,	0
AGGGGCGCAGGCGGGAGGGCGG	1,	0
AGGGGCGCAGGCGGGAGGGCGGCG	1,	1
AGGGGCGCAGGCGGGAGGGCG	0,	1
GAGGGCGGCGGGGCTGAGCGCGCCCTCCGTGC	1,	0
GGGGCTGAGCGCGCCCTCCGTGCTGCG	1,	0
CGCCCTCCGTGCTGCGTCCCTCG	12,	4
GCCCTCCGTGCTGCGTCCCTCG	11,	1
GCCCTCCGTGCTGCGTCCCTC	2,	3
CCCTCCGTGCTGCGTCCCTCGCGC	0,	1
CCCTCCGTGCTGCGTCCCTCGC	0,	1
CCCTCCGTGCTGCGTCCCTCG	0,	2
CCCTCCGTGCTGCGTCCCTCGCG	0,	1

chr17 46720006-46720028

GCTGCTGACCGGCTCGGCGACTGCCTCCCTGCTGTGAGCAGGAGAACAGGAAGTCTGCCCGACAGGGAGGTGGCCGGGCGGGAGCGGC

((((((..((.((((<<<---<<<<<<<<<<<-<<-<-<<(((...........)))))))).))))))))))))))))).))))))))

CGGCGACTGCCTCCCTGCTGTGAGCA	1, 0
CGGCGACTGCCTCCCTGCTGTGAGC	0, 3
CGGCGACTGCCTCCCTGCTGTGAG	0, 1
GGCGACTGCCTCCCTGCTGTGAGC	51, 29
GGCGACTGCCTCCCTGCTGTGAGCA	6, 1
GGCGACTGCCTCCCTGCTGTGAG	4, 0
GGCGACTGCCTCCCTGCTGTGA	2, 0
GCGACTGCCTCCCTGCTGTGAGC	9, 89
GCGACTGCCTCCCTGCTGTGAGCA	8, 21
GCGACTGCCTCCCTGCTGTGAGCAG	0, 2
CGACTGCCTCCCTGCTGTGAGCAG	0, 1
ACTGCCTCCCTGCTGTGAGCA	2, 0
AGGAGAACAGGAAGTCTGC	0, 1
GGAGAACAGGAAGTCTG	1, 9
TGCCCGACAGGGAGGTGGCCG	2, 0
TGCCCGACAGGGAGGTGGCCGG	1, 0
GCCCGACAGGGAGGTGGCCG	1, 0
CCCGACAGGGAGGTGGCCGGG	2, 0

chr2 109870426-109870447

AAATGTATTGGGAATGGGGCCTGGCCCCTGAGATGTGTAGGTTATACATCATACAACTCAGGGGCCAGGCACCATTCACATCACATTT

((((((..((.(((((<-<<<<<<<<<<<<<<-<<<<<.(((.....)))))))).)))))))))))))).)))))).))..))))))

GGGCCTGGCCCCTGAGATGTGT 5, 0

ACAACTCAGGGGCCAGGCACCA 0, 1

CAACTCAGGGGCCAGGCACCATT 0, 1

chr20 50733192-50733213

GCAAATTATGCTGTTGGGCATATGTGTATATATGTGTATGTGTATATATGTGTATGTACACATATGCCTAACAGCATAATTTGT

(((((((((((((<<<<<<<<<<<<<<<<<<<<<<-.((((....))))..)}}}}}}}}}}}}}}}}}}}}})))))))))))))

TTGGGCATATGTGTATATATGT 13, 1

TTGGGCATATGTGTATATATGT 13, 1

TTGGGCATATGTGTATATATGT 13, 1

TTGGGCATATGTGTATATATGT 13, 1

GTATGTACACATATGCCTAACA 12, 0

GTATGTACACATATGCCTAACA 12, 0

GTATGTACACATATGCCTAACA 12, 0

GTATGTACACATATGCCTAACA 12, 0

GTATGTACACATATGCCTAAC 2, 0

GTATGTACACATATGCCTAAC 2, 0

GTATGTACACATATGCCTAAC 2, 0

GTATGTACACATATGCCTAAC 2, 0

chr3 15124127-15124147

TGCTGTGGGGGTCATTGCTGCCTGTTGGTGTTGTGGTTACAGACAAGCTCACCAGTGCCAGCAAGGAGTCCCTGAGCACCCACACCA

((.(((((((-<<<--<<<-<<<<<<<<<--<(((((((.......))).)).)))..))))).)))))))...))).).)))))).))

GTCATTGCTGCCTGTTGGTGT 10, 2

GTCATTGCTGCCTGTTGGTGTTG 2, 2

GTCATTGCTGCCTGTTGGTGTTGT 0, 1

TCATTGCTGCCTGTTGGTGTTG 2, 2

CTCACCAGTGCCAGCAAGGAGTC 1, 2

CTCACCAGTGCCAGCAAGGAGT 0, 1

chr13 115010016-115010036

TATTAGTTTGGTGCAAAAGTAATTGCAGTTTTTGCTGTTGAACGTAGTGGTAAAACTGCAGTTATTTTTGCACCAACCTAATA

(((((.(((((((((((((((((((((((((.((..(((....)))..)>>>>>>>>>>>>>>>>>>>>>))))).))))))

GCAAAAGTAATTGCAGTTTTTGC 11, 0

CAAAAGTAATTGCAGTTTTTG 4, 2

CAAAAGTAATTGCAGTTTTTGC 3, 0

CAAAAGTAATTGCAGTTTTT 1, 0

AAAAGTAATTGCAGTTTTTGC 7, 0

AAAAGTAATTGCAGTTTTTGCT 4, 0

AAAAGTAATTGCAGTTTTTG 2, 0

TAAAACTGCAGTTATTTTTGC 17, 1

TAAAACTGCAGTTATTTTTGCA 8, 1

AAAACTGCAGTTATTTTTGC 1, 0

chr1 82174875-82174896

TCATTAGGGTGGTATAAAAGTAATTGTGGTATTTGCCATTAAAGTACTGCAAAAGCCGAAATTACTTTTGCACCAACCTAATGG

((((((((.((((.<<<<<<<<<<<<-<<<<-<<<<<.............))}}}=}}}}=}}}}}}}}}}}=})))).))))))))

TAAAAGTAATTGTGGTATTTGC 12, 61

AAAAGTAATTGTGGTATTTGC 1, 1

AAAGTAATTGTGGTATTTGCC 1, 0

AAAAGCCGAAATTACTTTTGC 4, 0

AAAAGCCGAAATTACTTTTGCA 1, 0

ATTACTTTTGCACCAACCTAAT 1, 0

chr9 5007628-5007649

TTATTAGGTTGACGCTAAAGTAATTGCGTTTTTTACTCATACTTTAATGGCAAAAACCACAATTACTTTTGCACCAGTCTAATAA

(((((((..((..((-<<<<<<<<<<<-<-<<<<<<-<-(((......)))).))))).).))))))))))).))..))..)))))))

TAAAGTAATTGCGTTTTTTACT	6, 0
TGGCAAAAACCACAATTACTTTT	1, 0
GCAAAAACCACAATTACTTTTGC	6, 0
GCAAAAACCACAATTACTTTTG	4, 0
CAAAAACCACAATTACTTTTGC	41, 1
CAAAAACCACAATTACTTTTG	6, 1
AAAAACCACAATTACTTTTGCA	107, 2
AAAAACCACAATTACTTTTGC	86, 0
AAAAACCACAATTACTTTTGCAC	6, 0
AAAAACCACAATTACTTTTG	3, 0
AAAACCACAATTACTTTTGCAC	84, 1
AAAACCACAATTACTTTTGCA	17, 1
AAAACCACAATTACTTTTGC	3, 0
AAAACCACAATTACTTTTG	1, 0
AAAACCACAATTACTTTTGCACC	1, 0
AAACCACAATTACTTTTGCACC	14, 10
AAACCACAATTACTTTTGCAC	7, 0
AACCACAATTACTTTTGCACCA	5, 1
AACCACAATTACTTTTGCACC	3, 1
ACCACAATTACTTTTGCACCA	25, 1

```
                                                  ACCACAATTACTTTTGCACC          5,  2

chr17 43012023-43012044
 CTTCCTGTTAGGGGAAAAGTCCTGATCCGGGAACCCACAGCCCCGTTCCTGGGCTTCTCCTCTGTAGCCAGCCTCAGCCGAGCAGCTGCTGGAGATACTGACCAGGGGG
 (((((((--<<<<<<-<<<<<<-<<--<<((..........)))).))..)))))).))))))(((.(((((...(((......))))))))...)))....)))))))
      GTTAGGGGAAAAGTCCTGATCCG                                                                                     1,  0
       TTAGGGGAAAAGTCCTGATCCG                                                                                     7,  0
       TTAGGGGAAAAGTCCTGATCC                                                                                      2,  0
       TTAGGGGAAAAGTCCTGATCCGG                                                                                    1,  7
       TTAGGGGAAAAGTCCTGATCCGGG                                                                                   0,  1
        TAGGGGAAAAGTCCTGATCCGG                                                                                    5,  46
        TAGGGGAAAAGTCCTGATCCGGG                                                                                   1,  4
        TAGGGGAAAAGTCCTGATCCGGGA                                                                                  0,  1
                                           CGTTCCTGGGCTTCTCCTCTGT                                                 0,  3
                                           CGTTCCTGGGCTTCTCCTCTGTA                                                0,  1
                                             TTCCTGGGCTTCTCCTCTGTAG                                               0,  9
                                              TCCTGGGCTTCTCCTCTGTA                                                0,  1
                                              TCCTGGGCTTCTCCTCTGTAG                                               0,  1

chr1  63966764-63966785
```

GTAGGGACTGGATTCATTATTTTGCATGTGTATAACCAGTTACTGAAACAGATATCCACATGTAAAAAAATGAATCTAGTCCCTAC

(((((((({{{{{{{{{{={{{{{{{{{{{.(((.(..(((.....))).).))>->>>>>>>>>>>->>>>>>>>))))))))))

TGGATTCATTATTTTGCATGTG 29, 3

TGGATTCATTATTTTGCATGT 1, 0

GGATTCATTATTTTGCATGTGT 1, 0

ATTCATTATTTTGCATGTGTA 1, 0

TTCATTATTTTGCATGTGTATA 5, 0

TTCATTATTTTGCATGTGTAT 3, 0

TTCATTATTTTGCATGTGTATAACC 1, 0

TCCACATGTAAAAAAATGAATC 79, 97

TCCACATGTAAAAAAATGAAT 16, 2

TCCACATGTAAAAAAATGAATCT 2, 0

CATGTAAAAAAATGAATCTAGT 0, 1

chr1 118397433-118397454

AACTCCTAGGTTCGTGACCAGGGGAAATATCTGCTACCAGATTAGAGATTAAGGAAATCTTGTAGCAGGTATTTTCCCTGGTAGGGGTT

(((((((.........(((((((((((((((((((((.(((((............))>>>->>>>>>>>>>>>>>>>>>))))))))))

TCTTGTAGCAGGTATTTTCCCT 12, 14

TCTTGTAGCAGGTATTTTCCC 0, 4

CTTGTAGCAGGTATTTTCCCT 0, 2

```
chr19 57950479-57950500

 ACCCAGGTTTTAGTGGCTCCCTCTGCCTGCAGAATGGAAGACCACGTGCAGGTGAGGGTGGAGCCACCGATACCTGGGT

 ((((((((.(--<<<<<<<<<<<<<<<<<<(...(((....)))..))))))).)))).))))))))..).))))))))

          TTAGTGGCTCCCTCTGCCTGCA                                                    3,  27

          TTAGTGGCTCCCTCTGCCTGC                                                     0,  18

           TAGTGGCTCCCTCTGCCTGC                                                     1,  11

           TAGTGGCTCCCTCTGCCTGCA                                                    1,  6

           TAGTGGCTCCCTCTGCC                                                        0,  1


chr8  99405895-99405916

 CCAGCTCTGGTTCCCCAGCCTACTGGAGGATAAGAGGATATAAAGGTCTCTTATCCTCCAGTAGACTAGGGAGCCAGAGCTGG

 (((((((((((((((.((.((((((((((((((((((.........))))>>>>>>>>>>>>>>->>->>>>)))))))))))

              CCCAGCCTACTGGAGGATAAGA                                                    0,  5

               CCAGCCTACTGGAGGATAAGA                                                    2,  2

               CCAGCCTACTGGAGGATAAGAG                                                   0,  4

               CCAGCCTACTGGAGGATAAG                                                     0,  1

                                                  CTTATCCTCCAGTAGACTAGGG                1,  0

                                                  CTTATCCTCCAGTAGACTAGG                 1,  0

                                                  CTTATCCTCCAGTAGACTAGGGA               0,  1
```

TTATCCTCCAGTAGACTAGGGA	12,	3
TTATCCTCCAGTAGACTAGGGAG	6,	3
TTATCCTCCAGTAGACTAGGGAGC	0,	1
TATCCTCCAGTAGACTAGGGAGC	3,	4
TATCCTCCAGTAGACTAGGGAG	0,	1
ATCCTCCAGTAGACTAGGGAGC	1,	0

chr1 173804513-173804534

ACATGTGTGAGCCACCGCGCCCGGCCAATAGATTTGTTCTTAAAGAACCAATTCATTGGCTGGGCGCGGTGGCTCACGCCTGT

(((.(((((((((((((((((((((((((..(((.((((.....)))).))>-->>>>>>>>>>>>>>>>>>>))))))).)))

AATTCATTGGCTGGGCGCGGTG	0,	1
ATTCATTGGCTGGGCGCGGTGG	1,	0
TTCATTGGCTGGGCGCGGTGGC	5,	3
TTCATTGGCTGGGCGCGGTGG	0,	5
TGGCTGGGCGCGGTGGCTCACG	1,	0
GGGCGCGGTGGCTCACGCCTG	2,	0
GGCGCGGTGGCTCACGCCTGT	2,	0
GCGCGGTGGCTCACGC	1,	0

chr20 50733230-50733251

```
GCAAATTATGCTGTTGGGCATATGTGTATATATGTGTATGTGTATATATGTGTATGTACACATATGCCTAACAGCATAATTTGT
((((((((((((<<<<<<<<<<<<<<<<<<<<<<-.((((....))))..)}}}}}}}}}}}}}}}}}}}}}})))))))))))
             TTGGGCATATGTGTATATATGT                                                     13,  1
             TTGGGCATATGTGTATATATGT                                                     13,  1
             TTGGGCATATGTGTATATATGT                                                     13,  1
             TTGGGCATATGTGTATATATGT                                                     13,  1
                                                   GTATGTACACATATGCCTAACA               12,  0
                                                   GTATGTACACATATGCCTAACA               12,  0
                                                   GTATGTACACATATGCCTAACA               12,  0
                                                   GTATGTACACATATGCCTAACA               12,  0
                                                   GTATGTACACATATGCCTAAC                2,  0
                                                   GTATGTACACATATGCCTAAC                2,  0
                                                   GTATGTACACATATGCCTAAC                2,  0
                                                   GTATGTACACATATGCCTAAC                2,  0
```
