# Supplementary material for: Reducing ligation bias of small RNAs in libraries for next generation sequencing
Source: Silence. 2012 May 30;3:4. doi: 10.1186/1758-907X-3-4 (PMC3489589; doi:10.1186/1758-907X-3-4)
Supplement: Additional file 12 — Table S5. Oligonucleotide sequences used in during the study. List of oligonucleotides in 5' to 3' orientation. n = denegenerate nucleotide. r = RNA. Fl = Fluoroscein. Phos = Phosphorylated. rApp = adenylated. 3ddC and sAaMO = 3' blocking group. AmMC6 = 5' blocking group. Red shows regions of interest and capitals highlight nucleotide changes compared to control template. [file 1758-907X-3-4-S12.pdf]

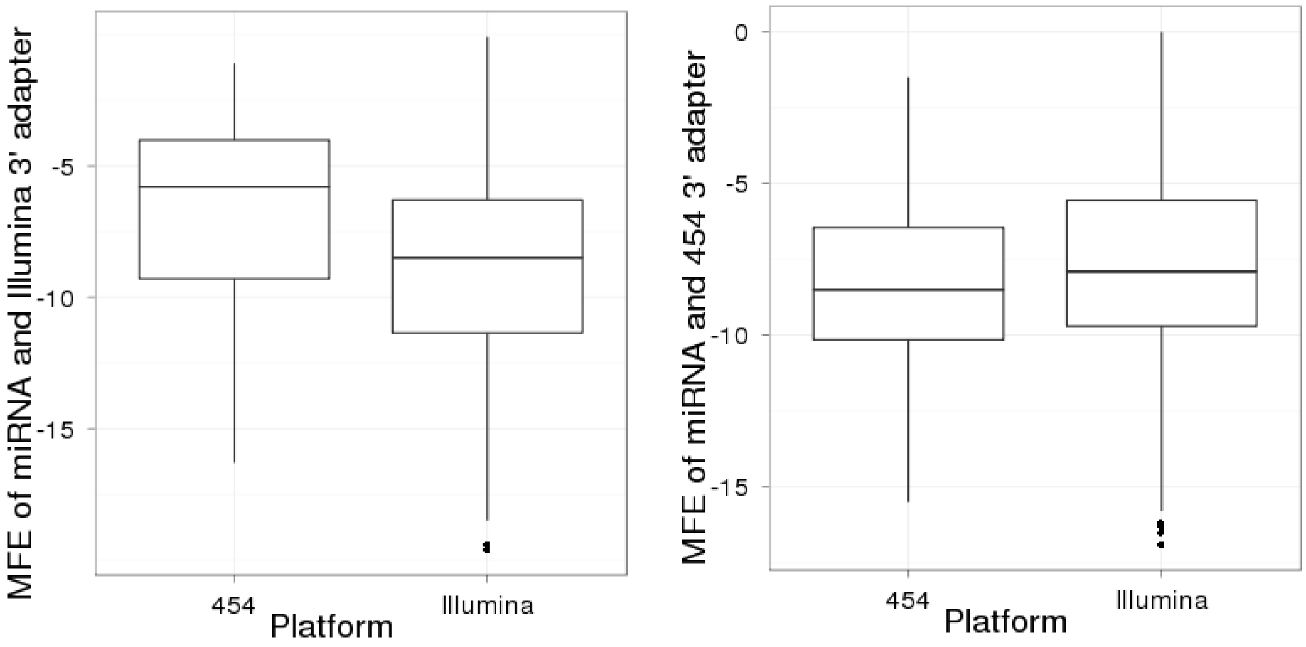

Supplementary Figure 7. Distributions of Minimum Free Energy (MFE) of known human miRNAs concatenated only with 3’ adapter sequences. Using Illumina adapter sequences the set of miRNAs found by Illumina has lower average MFE than the set of miRNAs found by 454 (left). Conversely, using 454 adapter sequences average MFE is lower for set of miRNAs found by 454 (right).
